# Exploring TB-Related Knowledge, Attitude, Behaviour, and Practice among Migrant Workers in Tajikistan

**DOI:** 10.1155/2011/548617

**Published:** 2012-01-19

**Authors:** Christopher Gilpin, Pierpaolo de Colombani, Sayohat Hasanova, Umrinisso Sirodjiddinova

**Affiliations:** ^1^International Organization for Migration, 1211 Geneva, Switzerland; ^2^Regional Office for Europe, World Health Organization, 2100 Copenhagen, Denmark; ^3^Country Office, World Health Organization, Dushanbe, Tajikistan; ^4^National TB Programme, Dushanbe, Tajikistan

## Abstract

A knowledge, attitude, behaviour, and practice survey was conducted among labour migrants in Tajikistan to elucidate key factors influencing access to tuberculosis diagnosis and care both in their labour destination country and at home. 509 labour migrants were interviewed in Khaton and Rasht Valley regions in Tajikistan using a standardised questionnaire. In addition, in-depth interviews were conducted among ten tuberculosis patients who had recently worked abroad. The study showed that migrants have increased vulnerability to tuberculosis due to the working and living conditions in the destination country and that access to health services is limited due to their legal status or the high cost of health services abroad. The average knowledge of migrants regarding tuberculosis is low and misconceptions are frequent. In Tajikistan, although tuberculosis drugs are usually provided free of charge, tuberculosis diagnosis and ancillary treatment are charged, thus creating a significant financial burden for patients and their families. Improving the access of labour migrants to affordable early diagnosis and treatment in both host countries and Tajikistan is a priority.

## 1. Introduction

Despite improvements in welfare since 2003, Tajikistan remains a country with widespread poverty. At the end of 2007, 53% of the population was poor and 17% was extremely poor (i.e., below the food poverty line) [1]. 75% of the poor live in rural areas (as do 71% of the extreme poor). The International Organization for Migration (IOM) identified that more than 620,000 Tajik citizens are labour migrants, with one in every four households in Tajikistan reporting a family member involved in labour migration [2]. These people primarily migrate not only to Russian Federation, but also to neighbouring Kazakhstan, Kyrgyzstan, and Uzbekistan. A vast majority of these people are temporary migrants and do return.

The latest estimation of tuberculosis (TB) incidence in Tajikistan is 206 cases per 100,000 population [3]. This incidence is the highest among the former Soviet Union countries and of the World Health Organization (WHO) European Region. 17% of the newly diagnosed and 62% of the previously treated TB cases in Tajikistan are estimated having multidrug resistance (MDR) TB, one of the highest rates in the world [4]. Tajik migrants are not only exposed to high levels of TB transmission in their home country and destination countries but also their act of migration and legal status often limit their access to health services.

Health risk factors are often linked to the legal status of migrants, determining the level of access to health and social services. Further contributors include poverty, stigma, discrimination, housing, education, social exclusion, gender, and differences in language and culture. TB is often associated with poor housing, malnutrition, and other factors which are commonly encountered by migrants who often find themselves at the lower end of the social strata. Migrants' health status can change after spending some time in their host environment, where they may be exposed to new pathogens, lifestyle, and social determinants that promote ill health such as TB.

Considering the above, The National Tuberculosis Programme of Tajikistan decided to undertake a knowledge, attitude, behaviour, and practice survey with the aim of elucidating the key factors influencing access to TB diagnosis and treatment among migrants in their labour destination country and home country. The survey has been supported financially by the United Nations Development Programme (UNDP), principal recipient of The Global Fund's TB grants received in Rounds 6 and 8, and executed in collaboration with International Organization for Migration (IOM), Research Centre SHARQ, and WHO.

## 2. Material and Methods

The survey was designed as a cross-sectional quantitative study among the labour migrant communities of Khatlon and Rasht Valley, the regions most affected by emigration in Tajikistan. The households to visit were sampled from the Research Center SHARQ's database of migrant households, which includes 3400 migrant households across the country and is updated annually. The minimum sample size was calculated of 345 households with 95% confidence interval, 5% precision, and 50% prevalence (chosen to yield the largest sample size given that study aimed to explore different characteristics with different frequencies). Migrant household was defined as household with a labour migrant of the over 15 years of age, who had lived and/or worked abroad during 2007-2008.

Thirty-six clusters were selected in Bokhtar, Vose, and Pyanj districts of Khatlon region and in Tajikabad and Tavildara districts of Rasht Valley region. 509 labour migrants were interviewed face-to-faceat their home using ad hoc developed questionnaire containing 48 multiple-choice questions exploring general and migration status, vulnerability abroad, health seeking behaviour abroad (for those 240 labour migrants who fall sick), knowledge of the disease (signs and symptoms, transmission, treatment), attitude towards TB (health seeking behaviour, confidentiality, stigma), and sources of TB information. The questionnaires were firstly tested with a small number of people and eventually administered by 17 trained interviewers in Russian, Tajik, and Uzbek languages as preferred by the respondent. Each of them was informed of the objectives of the study and asked to provide his/her consent at the beginning of the interview. Questionnaires were encoded to preserve confidentiality. Only 18 persons met eventually refused to participate in the survey.

In addition to interviewing labour migrants, the survey was enriched by a second, qualitative part based on in-depth interview of current and former TB patients who have been living and working outside Tajikistan during the previous two years. Interviews were stratified by location, with half of patients currently on intensive phase and half on continuation phase of TB treatment. Patients were identified through records at TB hospitals and TB dispensaries and sampled as the following: 3 patients from the Oblast TB Hospital in Kulyab, 4 from the Republican TB Hospital in Machiton (currently named as Shifo town), and 3 from the TB Dispensary in Dushanbe. All patients were interviewed by trained interviewers and after patient's informed consent. A checklist of 26 open-ended questions, adapted from a similar study conducted in Uzbekistan by Project HOPE [5], was used to explore personal experiences in accessing TB diagnosis and treatment in both destination country and home country. The interviews were conducted in Tajik and Russian languages (depending from the preference of the respondent) and took between one and one-and-half hour time each. They were transcribed in Tajik and Russian languages and subsequently translated into English. Respondents were remunerated for the time spent.

The 509 labour migrants were interviewed from 17 April to 5 May 2009 and the 10 TB patients from 4 to 18 June 2009. The ethical approval for this study was obtained from the Ministry of Health of Tajikistan.

The quantitative data collected by the survey were processed through SPSS software. Aberrant entries were cleaned and aggregated data tabulated. Distributions of characteristics describing the study respondents were examined and tabulated. Categorical data were described using frequencies and percentage. Continuous variables were converted into categorical variables to simplify the analysis. Aggregated variable “sufficient TB knowledge” was created, which was defined as citing correctly at least 3 TB signs and symptoms, airborne transmission and way of treatment (medications prescribed by doctor and completing the full course of treatment). Possible associations between sociodemographic characteristics of respondents and their knowledge, attitude, behaviour, and practice were examined using chi squared test of association of proportions and chi squared test of linear trends of proportions. The qualitative data collected from the in-depth interviews were analyzed by trying to identify individual experiences in accessing TB services and identify key features in a matrix.

## 3. Results

### 3.1. Profile of the Labour Migrants

Of the total 509 labour migrants interviewed, 99% were male. 39% aged 18–29 years, 34% aged 30–39 years, 21% aged 40–49 years and 6% older than 50 years.

Only 11% of migrants had only elementary education or no schooling, while 73% completed the secondary school education and 16% had higher education. Only 33% of the migrants were having an employment in Tajikistan at the time of the survey. Regarding the financial position of their family, 8% describing it as unable to buy sufficient food, 45% declared being able to buy food but clothes with difficulty, 39% able to buy both food and clothing but not more expensive items, 7% able to buy more expensive items such as a television or refrigerator, and 1% able to buy what they wanted. 21% of migrants were living 10 kilometres or more far from the nearest health centre.

The majority of migrants, that is, 54%, had returned to Tajikistan in the previous year, 36% more than one year ago and 10% many years ago. The main reason for returning to Tajikistan was family reasons (44%), followed by having lost the job (20%), job no longer available (13%), health reasons (10%), deportation back to Tajikistan (6%), global financial crisis (4%), and other reasons (3%). The majority of respondents (61%) has being working abroad for two or more years, 31% for one year and 8% for less than six months.

### 3.2. Vulnerability Abroad

**Figure 1:**
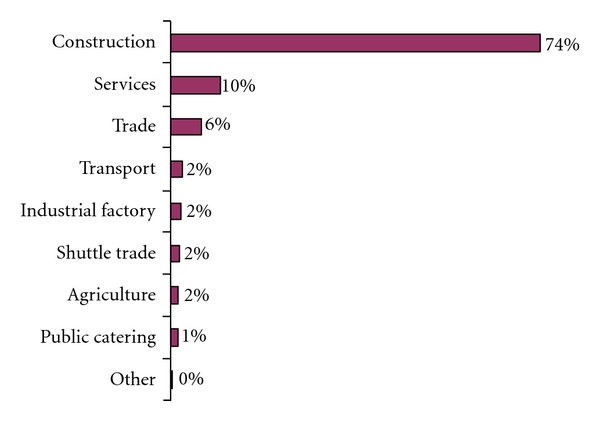
Type of employment abroad.

504 (99%) of the labour migrants had been working in Russian Federation. Differently from their economic situation in Tajikistan, migrants indicated a monthly income received abroad ranging from 58 to 1000 USD, with the following distribution: 7% earned 200 USD or less, 73% between 200–500 USD, and 7% more than 600 USD per month. At the time of the survey, the average monthly household income in Tajikistan was corresponding to 44 USD [1]. 379 (74%) of the labour migrants worked in the construction industry (see Figure 1).

Eighty percent of the migrants had obtained a work permit. Only 53% had an employment medical screening performed.

In terms of housing abroad, only 12% of the labour migrants have lived in own apartment or room, while the rest of them had shared accommodation: 24% shared a room with up to 4 persons, 20% shared a room with 4–8 persons, 9% shared room with more than 8 persons, 4% rest in a dormitory, 24% in the workplace or construction site and 7% in railroad cars.

In terms of nutrition, 88% of the labour migrants had adequate nutrition (defined as three meals a day) while working abroad; 8% had two meals per day and 4% had only one meal per day.

Twenty percent of the migrants reported to have had contact with a person with TB.

### 3.3. Health-Seeking Behaviour Abroad

Only 47% of the migrants indicated their employer providing social guarantees or benefits in case of illness (sick leave, paid medical services). For 76% of these migrants, medical services were actually paid. Only 24% of the migrants interviewed were entitled to paid sick leave.

Of the 509 migrants interviewed, 283 (56%) felt having limited opportunities to access medical services. The biggest felt obstacle for them was the high costs (77%), followed by the fear of losing the job if the employer knowing having an infection disease (8%), long distance to health facilities (5%), ignorance on where to go (4%), language (3%), fear of the medical staff (2%), unspecified (1%).

240 (47%) of the migrants interviewed became ill while working abroad. Of them, 160 (67%) self-medicated, 73 sought medical treatment, and 7 did not seek any treatment. Of those who had sought medical treatment abroad, 42 had to pay for the services received, 17 received free-of-charge services, 2 were refused of a treatment in a state hospital because they were without a residence permit, and 12 did not provide any detail. More description about the health services received abroad is in Table 1.

**Figure 2:**
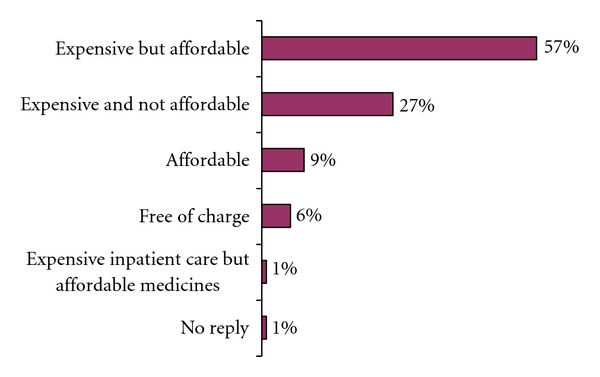
Perceived cost of medical services abroad.

The majority of the migrants (57%) who became ill while abroad found medical services and medicines expensive but affordable. Other answers are shown in Figure 2.

### 3.4. TB Knowledge and Awareness

Only 2 of the 509 migrants interviewed reported to have ever been diagnosed with TB and 74 (14%) had a family member diagnosed with TB any time in the past.

To the question suggesting most common signs and symptoms, the migrants characterized TB with loss of body weight (57%), cough (55%), cough with blood (47%), cough for 3 weeks and more (45%), fatigue (33%), fever (28%), shortness of breath (25%), chest pain (20%), nausea (19%), headache (16%), do not know (10%), and other (4%).

Sixty-three percent of migrants correctly knew that TB could be transmitted through the air when a person with TB coughs or sneezes. However, 70% of migrants also had the misconception that sharing dishes could transmit TB. Other misconceptions regarding TB transmission were as following: 66% by eating from the same plate, 19% through handshakes, and 14% from touching items in public places. 9% of migrants reported not to know how TB can be transmitted.

Eighty-three percent of migrants felt that TB can be cured. 69% migrants correctly knew that TB can be cured with specific drugs and 48% that completing all stages of treatment is necessary to cure TB. Misconceptions were also reported on how TB can be treated, such as with herbal remedies (25%), home rest without medicine (6%), religious treatment (3%), other (1%), and do not know (11%).

Only 100 (20%) of respondent were estimated to have “sufficient knowledge of TB” (see material and methods). “Sufficient knowledge” was weakly associated with level of education (*P* = 0.051): respondents with every higher level of education demonstrated better knowledge. “Sufficient knowledge” was found not associated with age, financial position of family, employment status, distance from health centre (Table 2), and perceived access to medical care abroad. Due to small number of female respondents, the study was not powered to detect any gender differentials.

Sixty-nine percent of migrants believed that anybody could get TB. Meantime, they also recognized that specific groups of population are at higher risk of TB, such as the poor (46%), homeless (24%), alcoholics (24%), drug users (12%), people living with HIV (17%) and persons who have been in prison (18%).

Regarding the TB services offered in Tajikistan, 34% of migrants did not know which ones are offered, 29% knew of directly observed treatment, 27% believed that TB medicines are free of charge, and 19% that TB diagnosis is free of charge.

Of the 509 migrants asked what are the top three sources to effectively reach people with information on TB, 78% indicated the television, 61% the health workers, and 36% the radio. Traditional written information, such as brochures and posters, were thought to be less effective (32%) than newspapers and magazines (25%). Information obtained from family, friends, neighbours, and colleagues was reported also effective source (32%).

### 3.5. TB Attitude and Health-Seeking Behaviour

Of the 509 migrants asked when seeking medical advice in case of TB symptoms, 24% replied they would wait for the symptoms to persist for 3-4 weeks, 14% would seek medical advice only after that self-treatment did not work, 14% would wait until they could no longer work and/or became very ill, and 3% would not go to a doctor.

**Figure 3:**
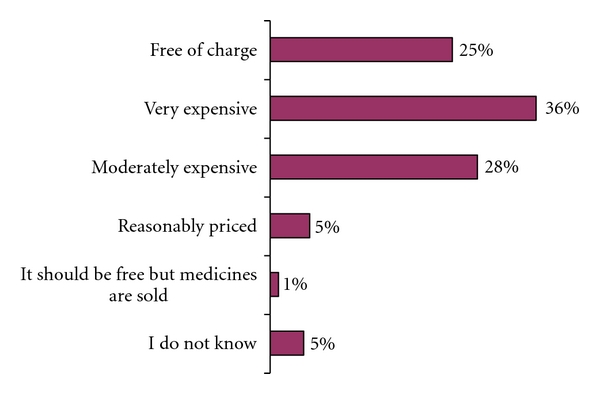
Perceived cost of TB services in Tajikistan

The perceptions of the migrants with regard to the cost of TB diagnosis and treatment in Tajikistan varied as shown in Figure 3.

Fifty-seven percent of the migrants declared to know a person who has or had TB. 39% of migrants indicated that the community mostly supports and helps persons with TB, 12% that community rejects persons with TB, 1% that community is indifferent and not supportive, and 1% did not know how their community would regard persons with TB.

With their attitude towards TB patients, all female respondents expressed compassion and willingness to support, resulting a statistically significant association between gender and attitude (*P* = 0.047). “Sufficient TB knowledge” was not associated with discriminating attitude towards TB patients, while level of education was associated in a linear pattern, where population with lower education had much positive attitude compared to those with higher education level (*P* = 0.046). In other words, discriminating attitude was the most commonly observed among the respondents of better education. Financial position of family, age, employment status and distance from nearest health centre were not related to discriminating attitude (Table 3).

### 3.6. Results from the In-Depth Interviews

Ten labour migrants (9 men and 1 woman) currently undergoing treatment for TB were interviewed more extensively. Their age ranged between 18 and 43 years, with an average of 28 years. 9 of these patients had pulmonary and one extra-pulmonary (abdominal) TB. At the time of the interview, 5 of them were in the intensive phase and 5 in continuation phase of treatment. Only one of them had medical insurance while working in Russia. All of them became sick while working in Russian Federation and had to return to Tajikistan, mainly for the high cost of the health services there (free of charge only for the Russian citizens), such as chest X-ray investigation and hospital treatment (around Russian Roubles 1000 per hospital day). The personal experience of each of these 10 returning labour migrants is reported below. Key features are summarized in Table 4.

**Patient 1**

A 32-year-old married man was first diagnosed with TB in 2005 in after his wife had already died of TB. He went to work in Russian Federation and became ill after five months. He returned to Tajikistan where he was diagnosed with TB and completed six months of treatment, of which three months in Machiton Hospital and three months at home. After his recovery he returned to work in Russian Federation but in 2009 became ill with the same symptoms and after two weeks of feeling ill returned to Tajikistan where he was diagnosed with TB and admitted to Machiton Hospital. Although satisfied with the treatment he was receiving in hospital, he indicated that doctors were too busy to answer questions about patients' treatment and could be more supportive. Brochures and booklets on TB are available for patients in the hospital. His family does suffer due to the burden of paying for health care and his inability to work.

**Patient 2**

A 25-year-old single man who had worked in Russian Federation for five years and at the end of 2008 became ill with coughing and weight loss. He did not seek medical care in Russian Federation due to the high cost of treatment and fear of deportation, returned home to Tajikistan where he was diagnosed with TB by chest X-ray and started treatment. He had been on treatment for six months in Machiton Hospital and not allowed to leave the hospital as the doctors had informed him that he was still infectious because of his delay in starting treatment. He felt the information provided to him regarding TB by nurses and from brochures was good. He felt that the condition of the hospital was not good and that the bathroom facilities were very bad. He was aware that he could be cured if he remained compliant and completed his treatment.

**Patient 3**

A 33-year-old married man who had lived and worked in Russian Federation for sixteen years together with his family. He was firstly diagnosed with TB in Russian Federation and spent there eight months receiving TB treatment in a private clinic. Treatment was expensive and the drugs received were mostly by intravenous infusions or injections. After spending all the money saved from his several years of work in Russian Federation, he had recently returned to Tajikistan and was on a five-drug treatment regimen including Streptomycin. He indicated that he would like to receive more information on TB and have discussions with his health providers on his treatment.

**Patient 4**

A 23-year-old single woman who had worked in Russian Federation for two years as a housemaid for a Russian family. She started to feel ill and returned to Tajikistan where was diagnosed with TB on X-ray examination and started treatment in March 2008. After a few days, she stopped taking medication due to nausea and headache but pretended to her own family to be compliant with treatment during two months. She returned to Moscow to continue her work but after one month she became ill again with fever, cough, and malaise. She applied for a factory job and diagnosed with TB through a medical screening in May 2009. She returned to Tajikistan and started a new treatment in Machinton Hospital. She is currently on a retreatment regimen including Streptomycin and understands the need to be compliant with treatment. She was happy with the information and support she was receiving in the hospital.

**Patient 5**

A 32-year-old married man who had worked for six years as a construction worker in Russian Federation where he was firstly diagnosed with TB colitis during a laparoscopic examination in December 2007. He returned to Tajikistan where he commenced six months TB treatment initially at home and then at Machiton Hospital and in Vose Health Centre. After two months from treatment completion, his conditions worsened, he had abdominal surgery in March 2009 restarted treatment at Kulab TB Dispensary. Although the TB drugs were provided free of charge, X-ray and sputum examinations, as well as intravenous solutions and vitamins, had to be paid and represented a financial burden for the patient. His family had to sell some livestock to cover the costs of his treatment. The hospital building in Kulab TB Dispensary has inadequate bathroom facilities located away from the main building. He was generally satisfied with the information he was receiving regarding his treatment but would like more regular education sessions from the health providers.

**Patient 6**

A 19-year-old man who had work two years as a guard for a construction project in Russian Federation. He was diagnosed with TB by X-ray there and returned to Tajikistan where the diagnosis was confirmed by sputum examination. He is receiving directly observed treatment three times a week in the Kulab Oblast TB Dispensary but refused inpatient care due to the poor hospital conditions. His family had to pay for vitamin supplements and the X-ray examinations. He is satisfied with the information being provided to him by health care workers.

**Patient 7**

An 18-year-old man who had worked for two years as a painter for a construction company in Russian Federation. He returned to Tajikistan after becoming sick and was diagnosed with TB by X-ray examination at Kulab TB dispensary in March 2009. He was hospitalized for the first two months of treatment and is currently continuing his treatment at home. He was satisfied with the information he was receiving from the health providers at the dispensary but said that this information was not given to him whilst in hospital. He was required to pay for all medication other than the TB drugs and also had to borrow money to pay for the cost of hospitalization and X-ray and sputum examinations. This was a financial burden for his family.

**Patient 8**

A 43-year-old divorced man who had worked for three years as an engine fitter in Russian Federation. He returned to Tajikistan in April 2009 where he was diagnosed with TB. He was admitted at Machiton Hospital for fifty days and is continuing treatment at home visiting the Dushanbe Dispensary for directly observed treatment three times a week. He preferred to have treatment at home due to the poor conditions of the hospital and the costs. He had spent all the money earned in Russian Federation on his treatment and suggested that all examinations and drugs should be free for patients.

**Patient 9**

A 27-year old married man who had worked 7-8 months every year for five years as a labourer for a construction company in Russian Federation. In February 2009, he was diagnosed with TB there but refused to start treatment due to its high cost (1000 Russian Roubles per day). He returned to Tajikistan two weeks later when his conditions worsened. Dushanbe TB Dispensary confirmed his diagnosis of TB and referred him to the Machiton Hospital for treatment but refused admission due to insufficient funds and is receiving four drugs from the Dispensary. The TB drugs are provided free of charge but he is required to buy vitamins and pay for other drugs and X-ray examinations. He was satisfied with the information he was being provided by the health providers in terms of interviews and brochures.

**Patient 10**

A 29-year old married man who had worked 8–10 months every year for six years as a labourer for a private construction company in Russian Federation. In February 2009, he was diagnosed with TB by chest X-ray there and commenced treatment. After 10 days of treatment in hospital he run away once he realized that the treatment cost 1200 Russian Roubles per day and that he would have to continue treatment for six months. He returned to Tajikistan where his TB diagnosis was confirmed. He refused treatment in hospital but was receiving free directly observed treatment three times a week at the Dushanbe TB Dispensary. However, he is required to pay for sputum smear and X-ray examinations and for vitamins. He had to borrow money to pay for these services. He was not satisfied with the information on TB he had received and would like to have access to booklets or brochures on TB.

## 4. Discussion

The study tried to explore TB-related knowledge, attitude, behaviour, and practice applied by a representative sample of Tajik labour migrants in their destination country, Russian Federation in almost all cases, and in Tajikistan. As expected [2], the large majority of them were found men, attracted to emigrate by better income opportunities offered in the construction industry, living and working abroad two or more years. The study confirms some of the findings of much larger study performed among Uzbek labour migrants in Kazakhstan [5].

The vulnerability to diseases and TB of Tajik migrants working in the Russian Federation is confirmed by the poor living and working conditions documented from the interviews of both quantitative and qualitative parts of the study. Although the majority of migrants interviewed had adequate nutrition in the destination country, they had to live in shared housing. Additionally, 20% reported to have had contact with a person with TB. Less than half of the migrants interviewed with questionnaire of in depth had the possibility to access to medical services paid by their employer and even less had the right to paid sick leave. However, almost half of them became ill when abroad. Of those who became ill, less then half sought medical treatment, which was described expensive or very expensive with the exception of some cases when services were provided free of charge. Of the 10 TB patients interviewed, only 2 started treatment in the Russian Federation and incurred in catastrophic expenditure because of the high costs. All others preferred to have TB treated in Tajikistan mainly because of this reason. The fear of accessing health care abroad, originating from the risk of deportation, was mentioned in both questionnaire and in-depth interviews.

From the in-depth interviews, it appears that Tajik labour migrants are travelling to Russian Federation for employment when healthy and only later become ill with TB there. It is not known whether Tajik labour migrants fall ill because of a reactivated latent TB infection acquired in Tajikistan or a reinfection from a recent contact with an active TB case in Russian Federation.

The survey clearly shows that a “sufficient” knowledge of TB is very low and misconceptions remain. “Sufficient” knowledge of TB seems only associated with higher levels of education but not with other socioeconomic characteristics of the migrants. Surprising, it resulted not leading to a better attitude towards TB and those with TB disease. Actually, higher level of education seems to worse this attitude and increase the stigma linked to TB. Television was recognized as the most important mean to disseminate TB information, followed by information from health workers and from the radio.

In Tajikistan, TB diagnosis and treatment are meant to be provided free of charge. However, the reality for the migrants interviewed with questionnaire or in depth was that sputum smear microscopy and chest X-ray investigation should be paid. Moreover, the needed ancillary treatment and vitamin supplements are also charged with significant financial burden to patients and their families. The poor conditions in the hospitals are main concern for the TB patients and many of them refused to be treated there. The provision of free ancillary treatment as well as social support to TB patients and their families are important to ensure adherence to treatment. The knowledge of the migrants with respect to available TB services in Tajikistan is also limited, especially on those services that should be provided free of charge.

Besides medical care, TB patients request more information about their condition to the hospital providers. None of the labour migrants interviewed and currently on TB retreatment regimens, mentioned drug resistant TB, which was likely to be their condition. This could be due to the inadequacy of laboratories in performing drug susceptibility testing, leading to under-diagnosis of drug-resistant TB in the country.

## 5. Conclusions

The findings suggest that TB is a significant problem among Tajik migrant communities and improving their access to affordable diagnosis and treatment in both Russian Federation and Tajikistan is a priority.

More awareness raising and education campaigns among migrant communities are needed. TB shows and spots on television should be considered first to increase awareness and deliver key health messages among population. However, other approaches can be also considered.

It will be important to advocate with migrant groups on the importance of undergoing screening for TB and other infectious diseases prior to departure. Consideration should be given to develop improved and affordable diagnostic services for TB to migrants prior to departure or upon return to Tajikistan. TB screening to migrants would also provide a unique opportunity to educate migrants on the signs and symptoms of TB and on the availability of TB services.

Additionally, it would be important to facilitate dialogue between the governments of both Tajikistan and Russian Federation on the importance of providing affordable diagnostic and treatment for TB among labour migrants regardless of their legal status. Specific strategies should be developed, national policies updated and intercountry collaboration ensured.

In Tajikistan, all services for diagnosis and treatment for TB patients should be made available free of charge, including the required ancillary treatment as well as the social support to TB patients, important measure to ensure treatment adherence, and social protection of their families to prevent catastrophic expenditures. Laboratory services and hospital care should be consistently improved, including the upgrading and renovation of facilities.

## Figures and Tables

**Table 1:** Health services received abroad.

Health service	*N*	%
*Free of charge services:*		
Hospital care paid by employer	7	12
Medical care by physician at place of work	3	5
Treatment from usual primary health care physician	3	5
State hospital care for a fracture	2	3
Dental care at state clinic	2	3
Subtotal	17	29

*Paid services (migrant perception of cost):*		
Hospital care in state hospital (very expensive)	15	25
Hospital care in private hospital (very expensive)	12	20
Tests performed in a polyclinic (very expensive)	7	12
Treated by ambulance paramedics (expensive)	6	10
Initial TB treatment before returning to Tajikistan	2	3
Subtotal	42	71

**Table 2:** Sufficient* TB knowledge associated with main sociodemographic characteristics of migrants.

Sociodemographic characteristics	Total sample (*n* = 509)		TB knowledge (*n* = 100)		*P*-value
	*n*	%	*n*	%	
Gender					
Female	6	1%	1	17%	0.853
Male	503	99%	99	20%	

Age groups					
18–29 years	200	39%	34	17%	0.464
30–39 years	173	34%	36	21%	
40 years and over	136	27%	30	22%	

Level of education					
No education or Primary	57	11%	8	14%	**0.051**
Secondary	373	73%	69	18%	
Tertiary	79	16%	23	29%	

Distance from the nearest health centre					
Less than 10 km	404	79%	83	21%	0.317
10 km and more	105	21%	18	17%	

Current employment status					
Employed	167	33%	38	23%	0.217
Unemployed	342	67%	62	18%	

Financial position of family (all 5 possible answers)					
Not enough money even to buy the food	41	8%	5	12%	0.414
Enough money for food but not for clothes	229	45%	45	20%	
Enough for food and clothes but not for expensive things	197	39%	44	22%	
We can buy some expensive things	35	7%	6	17%	
We can let us every thing we want	6	1%	0	0%	
No answer/refused to answer	1				

Financial position of family (answers grouped in 2 categories)					
Lower	270	53%	50	19%	0.481
Higher	238	47%	50	21%	
No answer/refused to answer	1				

Accessibility medical services abroad					
Yes	226	44%	51	23%	0.138
No	283	56%	49	17%	

* “Sufficient knowledge of TB” is defined as the capacity of correctly identifying at least three TB signs/symptoms (fever, fatigue, weight loss, cough, chest pain, shortness of breath), airborne transmission, and possibility to be cured by specific drugs taken without interruption.

**Table 3:** Attitude and stigma towards TB associated with main sociodemographic characteristics of migrants.

Sociodemographic characteristics	Total sample (*n* = 509)	Positive attitude (*n* = 308)		*P*-value
	*n*	*n*	%	
Gender				
Female	6	6	100%	**0.047**
Male	503	302	60%	

Age groups				
18–29 years	200	119	60%	0.368
30–39 years	173	100	58%	
40 and over	136	89	65%	

Level of education				
No education or Primary	57	37	65%	**0.046**
Secondary	373	233	62%	
Tertiary	79	38	48%	

Distance from the nearest health centre				
Less than 10 km	404	241	60%	0.438
10 km and more	105	67	64%	

Current employment status				
Employed	167	96	57%	0.717
Unemployed	342	212	62%	

Financial position of family (answers grouped in 2 categories)				
Lower	270	164	61%	0.880
Higher	238	143	60%	
No answer/refused to answer	1		0%	

Tuberculosis knowledge				
Sufficient knowledge	117	36	31%	0.426
Insufficient knowledge	392	64	16%	

**Table 4:** Summary of key features from in-depth interviews.

TB patient	Age/sex	Persons in household	Time spent in Russian Fed. before illness	Employment abroad	Income abroad (USD/month)	Living conditions abroad	Medical insurance or benefits abroad	Where TB diagnosed	Financial implications due to TB disease	Notes
1	32 years man	10	5 months	Construction worker in hospitals	~300	Shared room with 4-5 persons	Free treatment in hospital under construction	Tajikistan	Family suffers due to lost income and cost of treatment.	Felt sick two times in Russian Federation and returned to Tajikistan for treatment.

2	25 years man	4	5 years	Loader in supermarket	~660	Shared apartment with 8 persons	No insurance, no benefits	Tajikistan		Returned from Russian Federation after feeling sick (fear of deportation). Now, still infectious after 6-month treatment. Poor hospital conditions.

3	33 years man	28	16 years	Salesman in supermarket	~2000	Small house with wife and 3 children	Medical insurance	Russian Federation	Spent all money saved during 18 years working and living in Russian Federation.	8-month treatment in an expensive private clinic in Russian Federation. On retreatment regimen.

4	23 years women	8	2 years	Housemaid	~500	Shared apartment with 7 persons	Early 2009	Tajikistan		On retreatment regimen.

5	32 years man	8	6 years	Unskilled labourer in private company	~580	Shared apartment with 4 people	No insurance, no benefits	Russian Federation	Family had to sell livestock because of the cost of diagnostics, injections, and ancillary treatment.	On retreatment regimen. Poor hospital conditions.

6	19 years man	6	2 years	Guard in private construction company	Enough to live and travel back home	Shared room with 3-4 persons in the building under construction	No insurance, no benefits	Russian Federation	Family pays for X-ray examinations and vitamins.	Refused hospital treatment because in poor conditions.

7	18 years man	3	2 years	Painter in private construction company	~250	Shared room with 3 persons in the building under construction	No insurance, no benefits	Tajikistan	Needed to borrow money to pay X-ray and sputum examination, vitamin, and hospital.	Work of last months not paid because returned to Tajikistan.

8	43 years man	4	3 years	Engine fitter in private construction company	Enough to live and send some money home	Shared room with 12 persons	No insurance, no benefits	Tajikistan	Had spent all money earned in Russian Federation on his TB treatment.	

9	27 years man	4	5 years	Unskilled labourer in private company	~800	Shared room with 4 persons in the building under construction	No insurance, no benefits	Russian Federation	Had to pay X-ray, vitamin, and other drugs.	Diagnosed in Russian Federation, returned because of the expensive treatment there.

10	29 years man	8	6 years	Unskilled labourer in private company	~400	Barrack shared with 10 persons	No insurance, no benefits	Russian Federation	Needed to borrow money to pay chest X-ray and sputum examination, vitamin, and hospital.	Started treatment in Russian Federation, then returned because of the expensive treatment.
